# Exploring the impact of perioperative analgesia on postoperative chronic analgesic prescriptions in patients with lung cancer undergoing minimally invasive thoracic surgery: A retrospective observational study

**DOI:** 10.1002/ejp.4774

**Published:** 2024-12-27

**Authors:** Shizuha Yabuki, Yu Kaiho, Kunio Tarasawa, Saori Ikumi, Yudai Iwasaki, Takahiro Imaizumi, Kenji Fujimori, Kiyohide Fushimi, Masanori Yamauchi

**Affiliations:** ^1^ Department of Anaesthesiology and Perioperative Medicine Tohoku University Graduate School of Medicine Sendai Miyagi Japan; ^2^ Department of Health Administration and Policy Tohoku University Graduate School of Medicine Sendai Miyagi Japan; ^3^ Department of Emergency Medicine and Critical Care Medicine, Tochigi Prefectural Emergency and Critical Care Centre Imperial Foundation Saiseikai Utsunomiya Hospital Tochigi Japan; ^4^ Department of Advanced Medicine Nagoya University Hospital Nagoya Aichi Japan; ^5^ Department of Health Policy and Informatics Tokyo Medical and Dental University Graduate School of Medical and Dental Sciences Tokyo Japan

## Abstract

**Background:**

Lung cancer surgery is associated with a high incidence of chronic postsurgical pain (CPSP), which necessitates long‐term analgesic prescriptions. However, while essential for managing pain, these have shown various adverse effects. Current guidelines recommend using peripheral nerve blocks over epidural anaesthesia for perioperative analgesia in minimally invasive thoracic surgery (MITS). However, the impact of perioperative analgesia on chronic analgesic prescriptions remains unclear. Therefore, this study investigated chronic analgesic prescription patterns following MITS in patients with lung cancer who received either perioperative epidural anaesthesia or nerve block.

**Methods:**

We conducted a retrospective cohort study using data from the Japanese Diagnosis Procedure Combination database. Data were extracted from patients with primary lung cancer who underwent MITS between April 2018 and March 2022. Patients were divided into two groups based on the perioperative analgesia they received: the epidural anaesthesia group and the nerve block group. We compared the proportion of analgesic prescriptions 3–6 months postoperatively between both groups using multivariable logistic regression analysis. Inverse probability of treatment weighting was used to balance the covariates between the two groups.

**Results:**

Among the 38,719 eligible patients, 4513 (11.6%) were prescribed postoperative analgesics. We found no significant difference in the proportion of analgesic prescriptions between the epidural anaesthesia and nerve block groups (odds ratio, 1.00; 95% confidence interval, 0.99–1.01).

**Conclusions:**

This nationwide retrospective study suggests that the choice between perioperative epidural anaesthesia or nerve block in patients with lung cancer undergoing MITS does not influence the proportion of postoperative chronic analgesic prescriptions.

## 1 INTRODUCTION

Lung cancer surgery is among the most painful surgical procedures, with a high incidence of chronic postsurgical pain (CPSP) of 38.1% (Hazelrigg et al., 2002; Wang et al., 2023). CPSP is defined as chronic pain that intensifies after a surgical procedure and persists for at least 3 months (Schug et al., 2019). Despite a shift from traditional open thoracotomy to minimally invasive thoracic surgery (MITS), such as video‐assisted thoracoscopic surgery (VATS) and robot‐assisted thoracic surgery (RATS), the incidence of CPSP remains high, ranging between 25% and 35% (Chen et al., 2023; Klapper & D'Amico, 2015; Ujiie et al., 2019). CPSP negatively impacts activities of daily living and quality of life, leading to prolonged analgesic use (Brunes et al., 2020; Ishida et al., 2022; Ocay et al., 2020).

Long‐term analgesic prescriptions are associated with several challenges. For example, opioid use can lead to tolerance, dependence and misuse (Altawili et al., 2024). Opioids are also associated with adverse effects, including endocrinopathy, pain hypersensitivity and gastrointestinal issues (Gudin et al., 2015; Imam et al., 2018; Khan & Mehan, 2021). Similarly, non‐steroidal anti‐inflammatory drugs (NSAIDs) pose significant risks, such as gastrointestinal, renal and cardiovascular complications (Wehling, 2014). The complexity of managing chronic pain often leads to polypharmacy, raising concerns regarding drug interactions and additional side effects (Giummara et al., 2015). Furthermore, postoperative analgesics often require long‐term prescriptions (Yu et al., 2021; Zarling et al., 2016), imposing a significant economic burden (Beswick et al., 2018; Reuben et al., 2015; Turk, 2002). Consequently, this is an urgent public health issue that requires immediate resolution.

Effective perioperative pain management strategies may reduce the need for long‐term analgesics in CPSP (Althaus et al., 2012). CPSP was previously associated with neuroplastic changes, which were reduced after several days of postoperative regional anaesthesia (Sabanathan, 1995). Suggestively, a single‐shot nerve block probably cannot provide effective antinociceptive protection comparable to continuous epidural analgesia (Li et al., 2021). Moreover, achieving precise catheter placement for nerve blocks, particularly paravertebral blocks, is challenging due to the complexity of the paravertebral space (Luyet et al., 2009). Therefore, epidural anaesthesia may be more effective in preventing CPSP and could potentially reduce the need for chronic pain medication. Procedure‐specific postoperative pain management (PROSPECT) guidelines recommend peripheral nerve blocks over epidural anaesthesia for MITS. Peripheral blocks have comparable efficacy for perioperative pain management but are less invasive and associated with fewer complications (Feray et al., 2022). However, these guidelines provide limited insight into the impact of perioperative pain management strategies on CPSP (Yeap et al., 2020). Furthermore, the relationship between perioperative pain management during MITS and chronic analgesic prescriptions has yet to be investigated.

This study aimed to clarify the relationship between the choice of either perioperative epidural anaesthesia or nerve blocks and long‐term analgesic prescriptions following MITS in patients with lung cancer. In this exploratory study, we hypothesized that epidural anaesthesia would be associated with a lower proportion of chronic analgesic prescriptions than that of nerve blocks.

## 2 METHODS

### 2.1 Study design and ethics

This retrospective cohort study adhered to the Strengthening the Reporting of Observational Studies in Epidemiology (STROBE) statement (von Elm et al., 2008). This study was approved by the Institutional Review Board of Tohoku University (approval number: 2022–1‐444; August 2022). The requirement for informed consent was waived because of the anonymous nature of the data.

### 2.2 Data source

Patient data were extracted from the Japanese Diagnosis Procedure Combination (DPC) database, details of which have been previously described (Matsuda et al., 2011). This comprehensive database contains discharge summaries and administrative billing information from approximately 1100 acute‐care hospitals, covering approximately seven million patients annually and accounting for approximately 50% of all acute‐care hospital admissions in Japan. The DPC database archives demographic data, diagnoses coded using the 10th revision of the International Classification of Diseases and Injuries (ICD‐10), tumour‐node‐metastasis (TNM) classification of malignant tumours, comorbidities at admission, surgeries and procedures performed, and medication records. Outpatient data were collected from approximately the same number of hospitals that participated in the inpatient data collection. Outpatient data included diagnoses, procedures and medications (including outpatient prescriptions), and the extraction of diagnoses data has been possible since April 2018. Each hospital assigned inpatient and outpatient data unique anonymized IDs to link and track both types of data within the same institution.

### 2.3 Patient selection

From the DPC inpatient data, consecutive patients aged ≥18 years who were diagnosed with primary lung cancer (ICD‐10 codes C340, C341, C342, C343, C348 or C349) at a clinical stage of I or IIA and who underwent VATS or RATS were extracted between April 2018 and March 2022 (Table S1). Patients with stage IIB or higher were excluded because these stages include lung cancers with parietal pleura involvement, chest wall invasion and distant metastases, which may directly cause cancer‐related pain (Cappellari et al., 2018; Tsuzuki et al., 2023). Patients who underwent multiple surgeries during a single hospital stay, those who did not undergo surgery under general anaesthesia, those who did not receive either nerve block or epidural anaesthesia, or those who received both nerve block and epidural anaesthesia were excluded from the study. There is no clear guideline regarding the timing of follow‐up for patients after MITS, and variability in postoperative outpatient visit schedules was anticipated. Therefore, among the patients extracted from the inpatient cohort, we included only those with outpatient DPC data from 3 to 6 months postoperatively (Outpatients 3–6 M). We excluded those who had no visit records within 3–6 months postoperatively and those who were readmitted within 6 months postoperatively to eliminate the impact of hospitalization for additional lung cancer surgeries, chemotherapy, radiation therapy or other non‐lung cancer surgeries on the prescription of analgesics. To observe trends in the prescription of analgesics, we extracted data from patients with visit records from 0 to 3 months and 6–12 months postoperatively, following the same inclusion and exclusion criteria (Outpatients 0–3 M and Outpatients 6–12 M, respectively).

### 2.4 Exposure

The exposure variable in this study was epidural anaesthesia or nerve block use. The patients were divided into two groups: those who received general anaesthesia combined with epidural anaesthesia (epidural anaesthesia group) and those who received general anaesthesia combined with a nerve block (nerve block group). The technique for a nerve block or epidural anaesthesia was determined at the discretion of the anaesthesiologist or surgeon at each facility.

### 2.5 Outcomes

The primary outcome was the proportion of analgesic prescriptions among Outpatients 3–6 M, comparing the epidural anaesthesia and nerve groups. The secondary outcome was the proportion of analgesic prescriptions by type among Outpatients 0–3 M, Outpatients 3–6 M and Outpatients 6–12 M.

### 2.6 Outpatient prescription analgesics

Analgesics are classified into 12 categories according to the Guideline for the Pharmacologic Management of Neuropathic Pain 2nd edition (The Committee for the Clinical Guidelines of Pharmacotherapy for Neuropathic Pain, 2016) and Clinical Practice for Chronic Pain (The Committee for Clinical Practice Guideline for the Management of Chronic Pain, 2021), including NSAIDs, acetaminophen, weak opioids, strong opioids, pregabalin, mirogabalin, duloxetine, gabapentin, neurotrophins, antidepressants and antiepileptic drugs (Table S2). We excluded codeine phosphate as it can be prescribed for its antitussive effect (Oh et al., 2023), anxiolytics and muscle relaxants given their low level of recommendation in the Clinical Practice for Chronic Pain guidelines and traditional Chinese medicine due to the difficulty in identifying the specific symptoms targeted by the prescription. Prescribed analgesics were identified using the first seven digits of the 12‐digit National Health Insurance (NHI) drug code, as previously described (Higuchi et al., 2023). The 12‐digit NHI drug code is regulated by the Ministry of Health, Labor, and Welfare in Japan, where the first four digits indicate the therapeutic category and the next three digits represent the route of administration and ingredients.

### 2.7 Confounding variables

We extracted various patient baseline characteristics from the inpatient data, including age, sex, body mass index (BMI), smoking status (current/past smoker or non‐smoker), comorbidities at admission, type of surgery (VATS wedge resection, VATS segmentectomy, VATS lobectomy or RATS lobectomy), clinical cancer stages (TNM classification, 8th edition), Barthel Index at admission (Mahoney & Barthel, 1965), type of hospital (academic or non‐academic), intraoperative narcotic usage, duration of postoperative hospital stay (days), complications, total anaesthesia time, antithrombogenic drug (anticoagulants and antiplatelet agents) and analgesic usage at the time of admission. Age was divided into <60, 60–69, 70–79 and ≥80 years since 88.1% of patients extracted from inpatient data were aged ≥60. BMI was categorized according to the World Health Organization classification of <18.5, 18.5–24.9, 25.0–29.9 and ≥30.0 kg/m^2^ and missing values were treated as a separate category labelled ‘Missing’. For comorbidities at admission, we extracted ICD‐10 codes for hypertension, diabetes, heart failure, coronary artery disease, atrial fibrillation/atrial flutter, interstitial pneumonia, chronic obstructive pulmonary disease (COPD), cirrhosis, demyelinating disease, cerebrovascular disease and renal failure. The Charlson Comorbidity Index (CCI) (Charlson et al., 1987) was used to convert each ICD‐10 code into a score. Given that patients with malignant tumours are assigned two points in the CCI score, the minimum CCI score was 2. CCI scores were divided into three categories: 2, 3 and ≥ 4. The Barthel Index at admission was categorized as complete independence (100 points), partial assistance (45–95 points), assistance required (0–40 points) and ‘Missing’ (Mahoney & Barthel, 1965). Analgesics were defined as the same analgesic drugs as outpatient prescription analgesics. General anaesthesia time was categorized into 0–120, 121–240, 241–360 and ≥360 min. Missing values were found in both the Barthel Index and BMI, and they were handled as mentioned above. Therefore, all data were included in the analysis.

### 2.8 Statistical analyses

Continuous variables are presented as means and standard deviations, while categorical variables are presented as numbers and percentages. All statistical tests were two‐tailed. The primary outcome test was conducted at a significance level of 0.05, and statistical significance was set at *p* < 0.05. Standardized mean differences (SMDs) of baseline covariates between the epidural anaesthesia and nerve block groups were calculated, with an SMD within −0.10–0.10 indicating covariate balance (Austin, 2009).

The association between the choice of epidural anaesthesia or nerve block and outcomes was analysed using inverse probability of treatment weighting (IPTW) and logistic regression analysis. The following variables that could influence the outcomes and selection of epidural anaesthesia or nerve block were considered in the propensity score (PS) model: age, sex, BMI, TNM stage, type of surgery, CCI score, complications at admission, Barthel Index at admission, antithrombogenic drug usage and analgesic usage at the time of admission. After applying IPTW, the general anaesthesia time was incorporated into the logistic regression analysis with the selection of epidural anaesthesia or nerve block. Intraoperative narcotic usage was not included as a variable because it could be directly influenced by the choice of epidural anaesthesia or nerve block.

### 2.9 Subgroup analyses

Subgroup analyses were conducted based on the presence or absence of analgesic use at the time of admission, as determined by medical records. For those with prior analgesic usage, we defined the presence of an outpatient analgesic prescription as the situation where a new type of analgesic, different from the one brought, was prescribed. Bonferroni correction was applied to account for multiple comparisons, with *p‐*values <0.025 considered significant (i.e. 0.05/2).

### 2.10 Sensitivity analyses

We employed three additional approaches for sensitivity analysis. First, we use the instrumental variables (IV) method to address potential endogeneity issues within our model (Widding‐Havneraas & Zachrisson, 2022). The selection of epidural anaesthesia or nerve block mainly depends on the preference of anaesthesiologists and operators; therefore, we used the proportion of patients who underwent epidural anaesthesia among those who received VATS or RATS at each hospital as the IV. Using a two‐stage logit model for the IV model, we compared the outpatient prescription of analgesics between epidural anaesthesia and nerve block for perioperative analgesia. Using a partial F‐test, we verified the adequacy of the proportion of patients who underwent epidural anaesthesia among those who received VATS or RATS at each hospital as a strong instrument (F‐statistic >10) (Stock & Yogo, 2005).

Second, we repeated the analysis for Outpatients 6–12 M, following several previous studies that adopted a 6‐month postoperative CPSP evaluation period (Li et al., 2021; Wong et al., 2019).

Third, we redefined analgesics as NSAIDs, acetaminophen, weak opioids excluding codeine, strong opioids, pregabalin, mirogabalin and neurotrophins. Antidepressants and antiepileptic drugs were excluded since they could be prescribed for conditions other than pain.

Fourth, recognizing that strong opioids are typically prescribed for severe pain, we adopted a stricter definition by limiting analgesics to only strong opioids. The IPTW analysis was then performed based on the revised definition of analgesics. All statistical analyses were conducted using R version 4.3.2 (2023‐10‐31).

## 3 RESULTS

**FIGURE 1:**
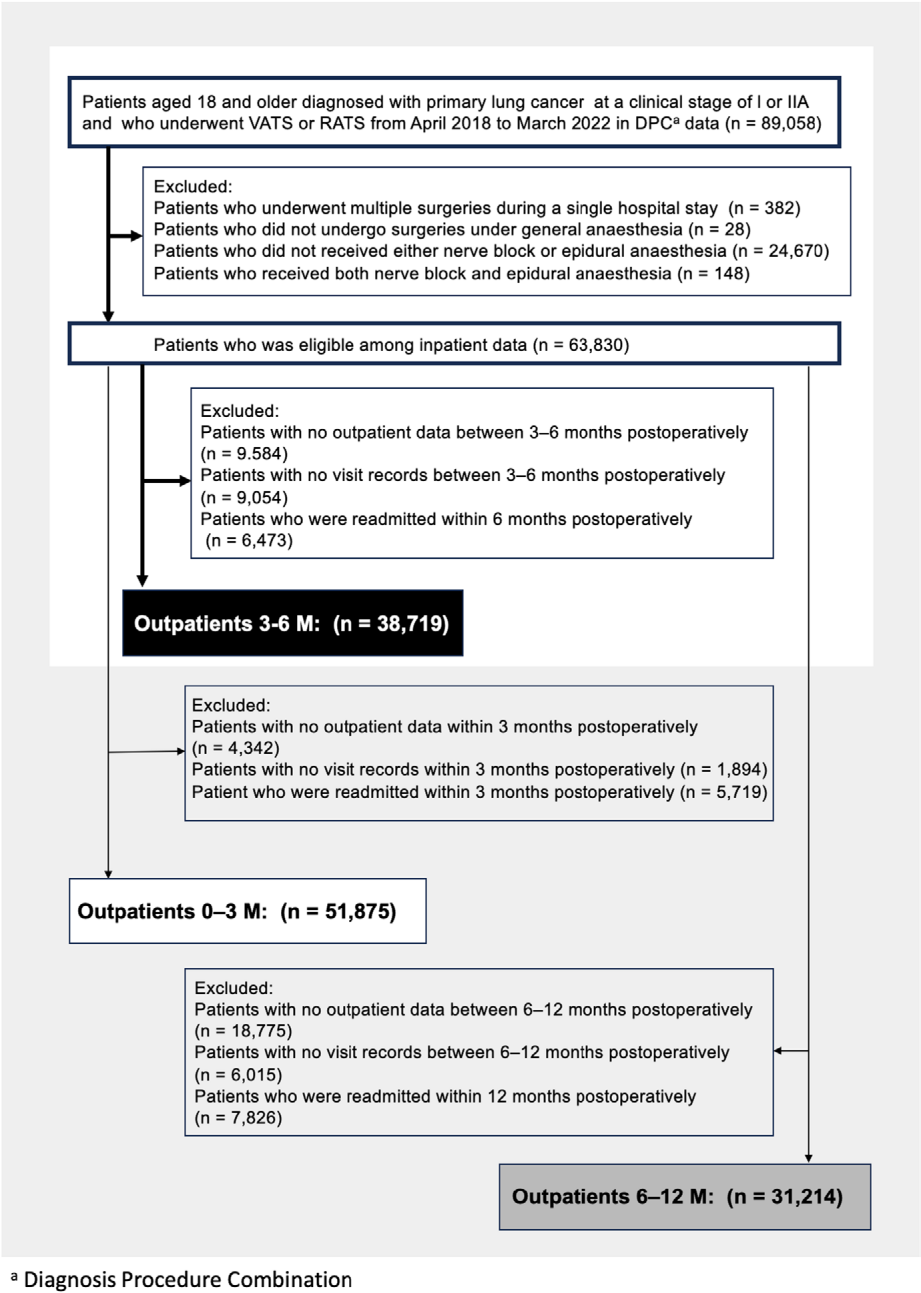
Flow diagram of patient selection.

A flow diagram of the study is shown in Figure 1. A total of 63,830 patients were eligible based on the data obtained during hospitalization. Among these, 38,719 (60.7%) patients were classified as Outpatients 3–6 M, comprising 31,670 patients in the epidural anaesthesia group and 7049 patients in the nerve block group. Similarly, 51,879 patients (81.3%) were extracted as Outpatients 0–3 M and 31,214 patients (49.0%) as Outpatients 6–12 M. Among the Outpatients 6–12 M, 28,048 patients (89.9%) matched with those in the Outpatients 3–6 M.

Patient baseline characteristics, hospitals, procedures and medications in the Outpatients 3–6 M before and after IPTW are shown in Table 1. Before IPTW, patients aged 70–79 years accounted for 48.9%, and females represented 43.9%. SMD analysis revealed no significant difference between the epidural anaesthesia and nerve block groups, with the exception of the type of operation, complications at admission and days of postoperative hospital stay. After IPTW, SMD analysis revealed that all variables in the epidural anaesthesia and nerve block groups were balanced. Demyelinating disease was excluded from the PS model due to the limited number of affected patients (epidural anaesthesia group, *n* = 0; nerve block group, *n* = 2).

**TABLE 1:** Baseline patient characteristics, hospitals, procedures and medications in the Outpatients 3–6 M cohort before and after IPTW.

		Outpatients 3–6 M													
		Before IPTW^a^							After IPTW^a^						
		All (*n* = 38,719)		Epidural Anaesthesia (*n* = 31,670)		Nerve Block (*n* = 7049)		Standard Mean Difference	All		Epidural Anaesthesia		Nerve Block		Standard Mean Difference
Age, *n* (%)															
<60		4576	(11.8)	3831	(12.1)	745	(10.6)	0.05	9236	(11.9)	4580	(11.8)	4655	(12.0)	−0.01
60–69		8506	(22.0)	7106	(22.4)	1400	(19.9)	0.06	16,993	(21.9)	8504	(22.0)	8488	(21.9)	0.00
70–79		18,936	(48.9)	15,436	(48.7)	3500	(49.7)	−0.02	37,795	(48.8)	18,930	(48.9)	18,864	(48.7)	0.00
≧80		6701	(17.3)	5297	(16.7)	1404	(19.9)	−0.08	13,432	(17.3)	6704	(17.3)	6727	(17.4)	−0.00
Sex (female), *n* (%)		16,995	(43.9)	14,038	(44.3)	2957	(42.0)	0.05	34,092	(44.0)	16,996	(43.9)	17,097	(44.1)	−0.01
Body mass index, kg/m^2^, *n* (%)															
<18.5		3151	(8.1)	2579	(8.1)	572	(8.1)	0.00	6285	(8.1)	3148	(8.1)	3138	(8.1)	0.00
18.5–24.9		25,290	(65.3)	20,761	(65.6)	4529	(64.3)	0.03	50,631	(65.4)	25,292	(65.3)	25,338	(65.4)	−0.00
25.0–29.9		8993	(23.2)	7321	(23.1)	1672	(23.7)	−0.01	17,959	(23.2)	8991	(23.2)	8968	(23.2)	0.00
30.0–34.9		1077	(2.8)	832	(2.6)	245	(3.5)	−0.05	2167	(2.8)	1078	(2.8)	1088	(2.8)	−0.01
≧35.0		134	(0.4)	110	(0.4)	24	(0.3)	0.00	269	(0.4)	133	(0.4)	136	(0.4)	−0.00
Missing		74	(0.2)	67	(0.2)	7	(0.1)	0.03	143	(0.2)	74	(0.2)	69	(0.2)	0.00
Charlson Comorbidity Index, *n* (%)															
2		21,218	(54.8)	17,517	(55.3)	3701	(52.5)	0.06	42,435	(54.8)	21,212	(54.8)	21,222	(54.8)	0.00
3		11,886	(30.7)	9.697	(30.6)	2189	(31.1)	−0.01	23,713	(30.6)	11,884	(30.7)	11,829	(30.5)	0.00
≧4		5615	(14.5)	4456	(14.1)	1159	(16.4)	−0.07	11,308	(14.6)	5623	(14.5)	5684	(14.7)	−0.00
Smoking, *n* (%)		22,991	(59.4)	18,652	(58.9)	4339	(61.6)	−0.05	45,828	(59.2)	22,983	(59.4)	22,845	(59.0)	0.01
Barthel index at admission, *n* (%)															
100		37,020	(95.6)	30.261	(95.9)	6659	(94.5)	0.07	74,061	(95.6)	37,022	(95.6)	37,040	(95.6)	0.00
95–45		1047	(2.7)	765	(2.4)	282	(4.0)	−0.09	2085	(2.7)	1047	(2.7)	1039	(2.7)	0.00
40–0		39	(0.1)	29	(0.1)	10	(0.1)	−0.02	73	(0.1)	38	(0.1)	34	(0.1)	−0.00
Missing		613	(1.6)	515	(1.6)	98	(1.4)	0.02	1236	(1.6)	613	(1.6)	623	(1.6)	0.00
Cancer stage, *n* (%)															
Stage 0		1988	(5.1)	1581	(5.0)	407	(5.8)	−0.03	3992	(5.2)	1988	(5.1)	2004	(5.2)	−0.00
Stage I		35,443	(91.5)	28,994	(91.6)	6449	(91.5)	0.00	70,895	(91.5)	35,445	(91.5)	35,450	(91.5)	0.00
Stage IIA		1288	(3.3)	1095	(3.5)	193	(2.7)	0.04	2569	(3.3)	1288	(3.3)	1282	(3.3)	0.00
Type of operation, *n* (%)															
VATS^b^ wedge resection		6339	(16.4)	4897	(15.5)	1442	(20.5)	−0.13	12,697	(16.4)	6342	(16.4)	6355	(16.4)	−0.00
VATS^b^ segmentectomy		5976	(15.4)	4718	(14.9)	1258	(17.8)	−0.08	11,929	(15.4)	5974	(15.4)	5956	(15.4)	0.00
VATS^b^ lobectomy		24,799	(64.1)	20,917	(66.0)	3882	(55.1)	0.23	49,639	(64,1)	24,801	(64.1)	24,837	(64.1)	−0.00
RATS^c^ lobectomy		1605	(4.2)	1138	(3.6)	467	(6.6)	−0.14	3191	(4.1)	1602	(4.1)	1589	(4.1)	0.00
Complication at admission, *n* (%)															
Hypertension		9680	(25.0)	7590	(25.1)	1730	(24.5)	0.01	19,292	(24.9)	9669	(25.0)	9623	(24.8)	0.00
Diabetes		7309	(18.9)	5857	(18.5)	1452	(20.6)	−0.05	14,605	(18.9)	7309	(18.9)	7295	(18.8)	0.00
Heart failure		1392	(3.6)	1077	(3.4)	315	(4.5)	−0.06	2730	(3.5)	1388	(3.6)	1342	(3.5)	0.01
Coronary artery disease		2361	(6.1)	1724	(5.4)	637	(9.0)	−0.14	4725	(6.1)	2364	(6.1)	2361	(6.1)	0.00
Atrial fibrillation/atrial flutter		2361	(6.1)	897	(2.8)	381	(5.4)	−0.13	2543	(3.3)	1279	(3.3)	1264	(3.3)	0.00
Interstitial pneumonia		1385	(3.6)	1126	(3.6)	259	(3.7)	−0.00	2742	(3.5)	1384	(3.6)	1358	(3.5)	0.00
Chronic obstructive pulmonary disease		2862	(7.4)	2401	(7.6)	461	(6.5)	0.04	5696	(7.4)	2862	(7.4)	2833	(7.3)	0.00
Cirrhosis		219	(0.6)	174	(0.6)	45	(0.6)	−0.01	437	(0.6)	219	(0.6)	218	(0.6)	0.00
Demyelinating disease		2	(0.0)	0	(0)	2	(0.0)	−0.02	–	–	–	–	–	–	–
Cerebrovascular disease		1385	(3.6)	1004	(3.2)	381	(5.4)	−0.11	2803	(3.6)	1392	(3.6)	1411	(3.6)	−0.00
Renal failure		745	(1.9)	539	(1.7)	206	(2.9)	−0.08	1488	(1.9)	748	(1.9)	740	(1.9)	0.00
Academic hospital, *n* (%)		10,182	(26.3)	8323	(26.3)	1859	(26.4)	0.00	20,474	(26.4)	10,190	(26.3)	10,283	(26.6)	−0.01
Oral medication at admission, *n* (%)															
Antiplatelets or anticoagulants		4279	(11.1)	3462	(10.9)	817	(11.6)	−0.02	8597	(11.1)	4282	(11.1)	4315	(11.1)	−0.00
One or more analgesics		3506	(9.1)	2883	(9.1)	623	(8.8)	0.01	7012	(9.1)	3504	(9.1)	3507	(9.1)	−0.00
Intraoperative narcotic usage															
Fentanyl, mean (SD^d^), mg		0.66	(0.6)	0.66	(0.6)	0.68	(0.6)	−0.03	–	–	–	–	–	–	–
Remifentanil, mean (SD^d^), mg		3.29	(2.0)	3.27	(2.0)	3.37	(2.0)	−0.05	–	–	–	–	–	–	–
Morphine, mean (SD^d^), mg		0.80	(8.2)	0.87	(8.5)	0.53	(6.6)	0.04	–	–	–	–	–	–	–
Ketamine, mean (SD^d^), mg		1.81	(17.7)	1.89	(18.7)	1.46	(12.6)	0.03	–	–	–	–	–	–	–
Postoperative hospital stay, mean (SD^d^), day		8.47	(0.5)	8.64	(9.7)	7.67	(8.4)	0.10	–	–	–	–	–	–	–
General anaesthesia Time, *n* (%)															
0–120 min		1960	(5.1)	1612	(5.1)	348	(4.9)	0.01	–	–	–	–	–	–	–
121–240 min		17,330	(44.8)	14,165	(44.7)	3165	(44.9)	−0.00	–	–	–	–	–	–	–
241–360 min		15,704	(40.6)	12,800	(40.4)	2904	(41.2)	−0.02	–	–	–	–	–	–	–
360 < min		3706	(9.6)	3076	(9.7)	630	(8.9)	0.03	–	–	–	–	–	–	–
Missing		19	(0.1)	17	(0.1)	2	(0.0)	0.01	–	–	–	–	–	–	–
One or more complication after surgery, *n* (%)		2523	(6.5)	1953	(6.2)	570	(8.1)	−0.08	–	–	–	–	–	–	–

^a^ Inverse probability of treatment weighting.

^b^ Video‐assisted thoracoscopic surgery.

^c^ Robot‐assisted thoracic surgery.

^d^ Standard deviation.

Logistic regression analysis revealed no significant difference in the proportion of analgesic prescriptions between the epidural anaesthesia and nerve block groups in Outpatients 3–6 M (odds ratio [OR], 1.00; 95% confidence interval [CI], 0.99–1.01) (Table 2).

**TABLE 2:** Results of logistic regression analysis in the Outpatients 3–6 M cohort, both unadjusted and following inverse probability of treatment weighting.

	Perioperative pain management	Proportion of analgesic prescription (%)	OR	95% CI	*p‐*value
Unadjusted	Epidural anaesthesia	11.7	1.032	0.95–1.12	0.445
	Nerve block	11.4	1	Reference	
After IPTW	Epidural anaesthesia	11.7	1.00	0.99–1.01	0.882
	Nerve block	11.6	1	Reference	

The proportion and type of analgesic prescriptions among patients across all postoperative periods are shown in Table 3. In total, 23,675 (45.6%) patients were prescribed analgesics as outpatients in Outpatients 0–3 M, 4513 (11.6%) patients in Outpatients 3–6 M and 3441 (11.0%) patients in Outpatients 6–12 M. Among these, NSAID usage was reported by 48.2% of Outpatients 0–3 M, 31.6% of Outpatients 3–6 M and 29.2% of Outpatients 6–12 M. Weak opioids were prescribed to 9.4% of Outpatients 0–3 M, 9.3% of Outpatients 3–6 M and 8.4% of Outpatients 6–12 M. Strong opioids were prescribed to 0.4% of Outpatients 0–3 M, 2.1% of Outpatients 3–6 M and 3.9% of Outpatients 6–12 M. Combined proportions of weak and strong opioids were prescribed to 4.4%, 1.2% and 1.3% of total patients across each period, respectively.

**TABLE 3:** Proportions and types of analgesic prescriptions among patients across each postoperative period.

	Outpatients 0–3 M	Outpatients 3–6 M	Outpatients 6–12 M
Total analgesic prescription	45.6%	11.6%	11.0%
NSAIDs	48.2%	31.9%	29.2%
Acetaminophen	24.5%	24.2%	27.1%
Weak opioids	9.4%	9.3%	8.4%
Strong opioids	0.4%	2.1%	3.9%
Pregabalin	11.8%	17.6%	14.2%
Mirogabalin	3.0%	4.9%	4.3%
Gabapentin	0.0%	0.1%	0.0%
Other antiepileptic drugs	0.8%	3.1%	3.3%
Duloxetine	0.7%	2.2%	2.5%
Tricyclic antidepressants	0.2%	0.8%	3.2%
Other antidepressants	0.5%	2.2%	2.2%
Neurotrophin	0.5%	1.5%	1.7%

Abbreviation: NSAIDs, Non‐steroidal anti‐inflammatory drugs.

The proportions of chronic analgesic prescriptions among patients with and without analgesic usage at the time of admission were 12.2% and 10.3%, respectively, in Outpatients 3–6 M. Subgroup analyses revealed no significant difference in the proportion of outpatient analgesic prescriptions between the epidural anaesthesia and nerve block groups (Table 4).

**TABLE 4:** Results of the logistic regression analysis for patients with and without prior analgesic usage in the Outpatients 3–6 M cohort following inverse probability of treatment weighting.

			Proportion of analgesic prescriptio*n* (%)	OR	95% CI	*p‐*value
Patients without analgesics at admission	Epidural anaesthesia		10.4	1.00	0.99‐–1.01	0.715
	Nerve block		10.2	1	Reference	
Patients with analgesics at admission	Epidural anaesthesia		12.0	0.99	0.96–1.02	0.616
	Nerve block		12.7	1	Reference	

The IV analysis assessing the impact of selecting either perioperative epidural anaesthesia or nerve block on postoperative analgesic prescriptions in Outpatients 3–6 M showed an F‐statistic of 19.9 (*p* < 0.001) (Table S3), suggesting that the proportion of patients who received epidural anaesthesia and underwent VATS or RATS at each hospital was an appropriate IV. No significant difference in the prescription of analgesics between the epidural anaesthesia and nerve block groups was observed (OR, 1.07; 95% CI, 0.94–1.22).

The patient baseline characteristics, hospitals and procedures in Outpatients 6–12 M for the epidural anaesthesia and nerve block groups before and after IPTW are shown in Table S4. SMD analyses showed that all variables in the two groups were balanced after IPTW. The results of the logistic regression analysis in Outpatients 6–12 M and redefined analgesics were similar to those in the main and IV analyses, whereby the selection of perioperative epidural anaesthesia or nerve block was not significantly associated with postoperative analgesic prescription (Tables S5–, S7).

## 4 DISCUSSION

This retrospective cohort study investigated the relationship between perioperative epidural anaesthesia or nerve block usage and postoperative long‐term analgesic prescriptions in over 38,000 patients with primary lung cancer who underwent MITS using data from a Japanese nationwide database. Multivariable logistic regression analysis after IPTW showed no difference in the proportion of analgesics prescribed in outpatients 3–6 months postoperatively between the two groups after adjusting for patient demographics, hospital factors and type of surgery, and remained consistent even in subgroup analyses based on the presence or absence of analgesic use at the time of admission. To the best of our knowledge, this study is the first in the world to examine the relationship between the choice of perioperative analgesia and chronic analgesic prescription following MITS in patients with lung cancer.

A systematic review reported an incidence of CPSP among patients who underwent VATS of 25.6%, with 56.5% of these patients requiring analgesics long‐term (14.5% of the total patients) (Wang et al., 2023). Another multi‐centre retrospective study in Japan reported an analgesic prescription rate of 16% and 9% at 3 and 6 months after lung cancer surgery including VATS, respectively (Sugiyama et al., 2018). Consistent with these findings, our study found that 11.6% of patients at postoperative 3–6 months and 11.0% of patients at postoperative 6–12 months required analgesic prescriptions.

Although a previous study showed that regional anaesthesia combined with general anaesthesia was associated with lower rates of long‐term analgesic prescription than general anaesthesia alone for various types of surgeries (Yu et al., 2021), no previous study has investigated the association between the choice of epidural anaesthesia or nerve block and chronic analgesic prescription in MITS. Some studies have examined the impact of epidural anaesthesia or nerve block on CPSP in thoracic surgeries, but results have been controversial. Most previous studies had limited sample sizes (20–50 patients per group) (Khoronenko et al., 2018; Zhao et al., 2023). CPSP definitions also vary across studies, including CPSP assessment timing (e.g. 4 weeks to 2 years after surgeries), pain assessment tools used (e.g. visual analogue scale, numerical rating scale [NRS] or original questionnaires), and the pain status and intensity for diagnosis (e.g. presence of pain at rest, pain while coughing or pain rated ≥ 4 on the NRS) (Khoronenko et al., 2018; Mori et al., 2022; Shaker et al., 2023; Wong et al., 2019; Yeap et al., 2020; Zhao et al., 2023). Moreover, most studies were limited to thoracotomy, with only two studies focusing on MITS (Yeap et al., 2020; Zhao et al., 2023). Our study, which focused on patients with lung cancer who underwent MITS, showed that there was no significant difference between the choice of epidural anaesthesia or nerve block and chronic analgesic prescription, using large sample size data obtained from multiple facilities across Japan. We defined chronic prescriptions as those prescribed 3–6 months postoperatively based on the definition from the International Association for the Study of Pain (Schug et al., 2019). We also extended the period for analgesic prescriptions over a longer duration as a sensitivity analysis, thereby enhancing the robustness of our findings.

Moreover, several previous studies excluded patients taking preoperative analgesics (Khoronenko et al., 2018; Yeap et al., 2020; Zhao et al., 2023), which may indicate a history of moderate‐to‐severe chronic pain (Gerbershagen et al., 2009; Yu et al., 2021). Patients with chronic pain develop central sensitisation from sustained pain stimulation of the peripheral nerves, leading to hyperalgesia and allodynia (Coderre et al., 1993; Jensen & Finnerup, 2014; Ji et al., 2018). Upon conducting a subgroup analysis based on the presence or absence of preoperative analgesic usage, we found no difference in the proportion of analgesic prescriptions during the chronic phase between the epidural anaesthesia and nerve block groups, even among those with prior analgesic use.

Although an analgesic prescription does not always indicate the presence of pain, prescriptions given during the chronic phase after surgical wounds have healed generally indicate the patient's need for pain relief (Yu et al., 2021). Investigating the presence or absence of analgesic prescriptions is an objective method that yields consistent results, especially when using data obtained from multiple facilities. Moreover, one report suggested that the cutoff value for requiring additional analgesics is set at an NRS score of 4, representing moderate‐to‐severe pain (Gerbershagen et al., 2011). Therefore, our results may indicate that there was no difference in the incidence of moderate‐to‐severe CPSP requiring analgesics between the two groups. In CPSP research, the subjective nature of pain, the need for questionnaires, phone interviews or face‐to‐face evaluations, and the requirement for long‐term follow‐up make it difficult to secure sufficient sample sizes (Simanski et al., 2014). Thus, focusing on analgesic prescriptions for CPSP and using large‐scale real‐world data could address this limitation.

Unlike reports from various countries where opioids were included in 77–100% of postoperative pain prescriptions (Hill et al., 2017; Kurteva et al., 2021), the proportion of weak and strong opioid prescriptions in this study was low (<5%). This may be attributable to the Japanese NHI system, which restricts the eligibility and duration of covered medications, or the tendency of Japanese physicians to be cautious when prescribing opioids for acute and chronic pain (Onishi et al., 2017).

This study had several limitations. First, the administrative claims data lacked detailed information on epidural anaesthesia and nerve blocks such as the specific type of nerve block used, the presence of catheter placement, the timing and duration of the procedures, as well as the types of local anaesthetics and additives and their effectiveness. Additionally, the data did not include information on other potential therapies, including physical and psychological interventions. Therefore, the influence of these factors on the results was not considered. However, our results reflect real‐world settings, including these treatments, owing to the large and nationwide database used. Second, the DPC database only connects inpatient and outpatient data within the same hospital. Thus, it was not possible to track patients who were transferred or referred to other hospitals, potentially leading to an underestimation of analgesic prescriptions. However, patients with cancer in Japan are generally followed‐up at the hospitals where they underwent surgery, as shown in a previous study linking inpatient and outpatient data from the DPC database (Makito et al., 2020). Third, as a retrospective observational study, there is a possibility of residual confounding. Therefore, we used the IPTW with propensity scores to balance the measurable variables between both groups, adjusting for possible confounding effects. Furthermore, IV analysis helped to mitigate the impact of unmeasured confounding effects. Fourth, this study is an exploratory, retrospective comparative analysis; therefore, it demonstrates associations rather than causation. Lastly, this study primarily investigated the relationship between perioperative pain management and postoperative analgesic prescriptions, rather than directly evaluating the severity of CPSP. Consequently, this study did not address the impact of perioperative pain management strategies on mild CPSP that does not require analgesics. Since even mild postoperative pain can affect patients' quality of life, further research is needed in this area.

## 5 CONCLUSIONS

This study revealed no significant difference in the proportion of chronic analgesics prescribed to patients with primary lung cancer who underwent MITS with either perioperative epidural anaesthesia or nerve block. Our findings may provide new insights into chronic analgesic prescriptions and inform the PROSPECT guidelines, which currently recommend nerve blocks as the first choice for perioperative pain management in MITS.

## AUTHOR CONTRIBUTIONS

Shizuha Yabuki designed this study. Shizuha Yabuki, Yu Kaiho, Saori Ikumi, Yudai Iwasaki and Takahiro Imaizumi contributed to the conceptualization. Kunio Tarasawa, Kenji Fujimori and Kiyohide Fushimi curated the original resources. Shizuha Yabuki, Yu Kaiho and Takahiro Imaizumi analysed the data. Shizuha Yabuki, Yu Kaiho and Yudai Iwasaki edited the original draft. Yu Kaiho, Kunio Tarasawa, Takahiro Imaizumi, Saori Ikumi, Yudai Iwasaki, Kenji Fujimori and Masanori Yamauchi revised the manuscript. All authors have approved the final version of the manuscript and agreed to be accountable for all aspects of the work.

## Supporting information


Table S1:

Table S2:

Table S3:

Table S4:

Table S5:

Table S6:

Table S7:

## Data Availability

Individual‐level data are not available due to agreements with the contributing hospitals and the approval conditions of the institutional review boards that approved this study. Additional anonymized summaries of the data supporting the present study, as well as the detailed study protocol, are available from the corresponding author upon reasonable request.
