# Supplementary material for: Exploring the impact of perioperative analgesia on postoperative chronic analgesic prescriptions in patients with lung cancer undergoing minimally invasive thoracic surgery: A retrospective observational study
Source: Eur J Pain. 2024 Dec 27;29(2):e4774. doi: 10.1002/ejp.4774 (PMC11680971; doi:10.1002/ejp.4774)
Supplement: Supplementary file 1 — Table S1: [file EJP-29-0-s003.docx]

Table S1. Type of surgeries as video-assisted thoracoscopic surgery (VATS) and robot-assisted thoracic surgery (RATS) based on Japanese administrative claims data

| Type of surgery | Billing code |
| --- | --- |
| VATS Wedge Resection | 150358610 |
| VATS Segmentectomy | 150358710 |
| VATS Lobectomy | 150358810 |
| RATS Lobectomy | 150406110 |
