# Supplementary material for: Exploring the impact of perioperative analgesia on postoperative chronic analgesic prescriptions in patients with lung cancer undergoing minimally invasive thoracic surgery: A retrospective observational study
Source: Eur J Pain. 2024 Dec 27;29(2):e4774. doi: 10.1002/ejp.4774 (PMC11680971; doi:10.1002/ejp.4774)
Supplement: Supplementary file 2 — Table S2: [file EJP-29-0-s001.docx]

Table S2. National Health Insurance (NHI) drug codes of prescription in each category: non-steroidal anti-inflammatory drugs (NSAIDs), acetaminophen, weak opioids, strong opioids, pregabalin, mirogabalin, duloxetine, gabapentin, other antiepileptic drugs, tricyclic antidepressants, other antidepressants, and neurotoropin.

| **Category** | NHI drug price standard listing code | |
| --- | --- | --- |
| NSAIDs | Flufenamate Aluminum | 1141004 |
|  | Mefenamic Acid | 1141005 |
|  | Sodium Salicylate | 1143009 |
|  | Aspirin | 1143010, 1149105, 1143700 |
|  | Indometacin | 1145001, 1145002, 1145005, 1145700 |
|  | Acemetacin | 1145003 |
|  | Proglumetacin Maleate | 1145004 |
|  | Diclofenac Sodium | 1147002, 1147700 |
|  | Amfenac Sodium Hydrate | 1147006 |
|  | Tiaramide Hydrochloride | 1148001 |
|  | Emorfazone | 1148004 |
|  | Ibuprofen | 1149001, 3399100, 1149702 |
|  | Ketoprofen | 1149005, 1149700 |
|  | Naproxen | 1149007 |
|  | Flurbiprofen | 1149011 |
|  | Sulindac | 1149015 |
|  | Fenoprofen | 1149016 |
|  | Piroxicam | 1149017, 1149701 |
|  | Loxoprofen Sodium Hydrate | 1149019 |
|  | Lobenzarit Sodium | 1149020 |
|  | Tenoxicam | 1149021 |
|  | Alminoprofen | 1149022 |
|  | Tiaprofenic Acid | 1149025 |
|  | Oxaprozin | 1149026 |
|  | Nabumetone | 1149027 |
|  | Zaltoprofen | 1149029 |
|  | Ampiroxicam | 1149030 |
|  | Etodolac | 1149032 |
|  | Mofezolac | 1149033 |
|  | Meloxicam | 1149035 |
|  | Lornoxicam | 1149036 |
|  | Celecoxib | 1149037 |
|  | Isopropylantipyrine | 1149100, 1149102 |
|  | Ethenzamide | 1149103 |
| Acetaminophen | 1141001, 1141007, 1149116, 1141700 | |
| Weak opioids | Pentazocine | 1149034 |
|  | Tramadol | 1149038, 1149117 |
|  | Buprenorphine | 1149703, 1149704 |
|  | Opium | 8112001, 8113001, 8114001, 8119100 |
| Strong opioids | Morphine | 8114002, 8114003, 8114004, 8114005, 8114700 |
|  | Pethidine | 8211001 |
|  | Oxycodone | 8119002 |
|  | Fentanyl | 8219001, 8219700, 8219701 |
|  | Methadone | 8219002 |
|  | Tapentadol | 8219003 |
| Pregabalin | 1190017 | |
| Mirogabalin | 1190026 | |
| Duloxetine | 1179052 | |
| Gabapentin | 1139007 | |
| Other antiepileptic drugs | Sodium Valproate | 1139004 |
|  | Phenytoin | 1132002, 1139100, 1139103, 1139104, 1139105 |
|  | Clobazam | 1139006 |
|  | Sultiame | 1137001 |
|  | Carbamazepine | 1139002 |
|  | Ethotoin | 1132001 |
|  | Acetylpheneturide | 1131001 |
|  | Zonisamide | 1139005 |
|  | Clonazepam | 1139003 |
|  | Ethosuximide | 1139001 |
|  | Topiramate | 1139008 |
|  | Acetylpheneturide | 1131001 |
|  | Trimethadione | 1133002 |
|  | Lamotrigine | 1139009 |
|  | Primidone | 1135002 |
|  | Levetiracetam | 1139010 |
|  | Stiripentol | 1139011 |
|  | Rufinamide | 1139012 |
|  | Perampanel Hydrate | 1139014 |
|  | Vigabatrin | 1139013 |
|  | Lacosamide | 1139015 |
|  | Mystan | 1139006 |
|  | Midazolam | 1139700 |
| Tricyclic antidepressants | Amitriptyline Hydrochloride | 1179002 |
|  | Nortriptyline Hydrochloride | 1179004 |
|  | Imipramine Hydrochloride | 1174006 |
| Other antidepressants | Amoxapine | 1179001 |
|  | Trimipramine Maleate | 1174005 |
|  | Clomipramine Hydrochloride | 1174002 |
|  | Dosulepin Hydrochloride | 1179027 |
|  | Lofepramine Hydrochloride | 1174004 |
|  | Maprotiline Hydrochloride | 1179008 |
|  | Mianserin Hydrochloride | 1179033 |
|  | Setiptiline Maleate | 1179034 |
|  | Fluvoxamine Maleate | 1179039 |
|  | Paroxetine Hydrochloride Hydrate | 1179041 |
|  | Sertraline Hydrochloride | 1179046 |
|  | Escitalopram Oxalate | 1179054 |
|  | Milnacipran Hydrochloride | 1179040 |
|  | Mirtazapine | 1179051 |
|  | Vortioxetine Hydrobromide | 1179060 |
| Neurotoropin | 1149023 | |
