# Supplementary material for: Exploring the impact of perioperative analgesia on postoperative chronic analgesic prescriptions in patients with lung cancer undergoing minimally invasive thoracic surgery: A retrospective observational study
Source: Eur J Pain. 2024 Dec 27;29(2):e4774. doi: 10.1002/ejp.4774 (PMC11680971; doi:10.1002/ejp.4774)
Supplement: Supplementary file 3 [file EJP-29-0-s002.docx]

Table S4. Baseline patient characteristics, hospitals, procedures and medications in the Outpatients 6–12 M cohort before and after IPTW[a]

	Outpatients 6–12 M													
	Before IPTW[a]							After IPTW[a]						
	All (n = 31,214)		Epidural Anaesthesia (n = 25,620)		Nerve Block (n = 5,594)		Standard Mean Difference	All		Epidural Anaesthesia		Nerve Block		Standerd Mean Difference
Age, n (%)														
< 60	3,648	(11.7)	3,067	(12.0)	581	(10.4)	0.05	7,340	(11.8)	3,650	(11.7)	3,690	(11.8)	-0.00
60 - 69	6,859	(22.0)	5,778	(22.6)	1,081	(19.3)	0.08	13,683	(21.9)	6,856	(22.0)	6,826	(21.9)	0.00
70 - 79	15,261	(48.9)	12,464	(48.7)	2,797	(50.0)	-0.03	30,474	(48.8)	15,258	(48.9)	15,216	(48.7)	0.00
≧ 80	5,446	(17.5)	4,311	(16.8)	1,135	(20.3)	-0.09	10,949	(17.5)	5,450	(17.5)	5,499	(17.6)	-0.00
Sex (female), n (%)	13,670	(43.8)	11.332	(44.2)	2,338	(41.8)	0.05	27,430	(43.9)	13,671	(43.8)	13,760	(44.1)	-0.01
Body mass index, kg/m^2, n (%)														
<18.5	2,551	(8.2)	2,118	(8.3)	433	(7.7)	0.02	5,048	(8.1)	2,547	(8.2)	2,501	(8.0)	0.01
18.5-24.9	20,334	(65.1)	16,753	(65.4)	3,581	(64.0)	0.03	40,768	(65.3)	20,339	(65.2)	20,428	(65.4)	-0.01
25.0-29.9	7,316	(23.4)	5,940	(23.2)	1,376	(24.6)	-0.03	14,611	(23.4)	7,315	(23.4)	7,296	(23.4)	0.00
30.0-34.9	840	(2.7)	664	(2.6)	176	(3.2)	-0.03	1,671	(2.7)	841	(2.7)	830	(2.7)	0.00
≧35.0	108	(0.4)	85	(0.3)	23	(0.4)	-0.01	216	(0.4)	108	(0.3)	108	(0.4)	-0.00
Missing	65	(0.2)	60	(0.2)	5	(0.1)	0.04	132	(0.2)	65	(0.2)	68	(0.2)	-0.00
Charlson comorbidity Index, n (%)														
2	17,157	(55.0)	14,222	(55.5)	2,935	(52.5)	0.06	34,325	(55.0)	17,154	(55.0)	17,171	(55.0)	-0.00
3	9,567	(30.7)	7,823	(30.5)	1,744	(31.2)	-0.01	19,067	(30.5)	9,564	(30.6)	9,503	(30.4)	0.01
≧4	4,490	(14.4)	3,575	(14.0)	915	(16.4)	-0.07	9,054	(14.5)	4,497	(14.4)	4,557	(14.6)	-0.01
Smoking, n (%)	18,596	(59.6)	15,169	(59.2)	3,427	(61.3)	-0.04	37,035	(59.3)	18,589	(59.6)	18,446	(59.1)	0.01
Barthel Index at admission, n (%)														
100	29,816	(95.5)	24,537	(95.8)	5,279	(94.4)	0.07	59,641	(95.5)	29,816	(95.5)	29,824	(95.5)	0.00
95-45	860	(2.8)	628	(2.5)	232	(4.1)	-0.09	1,726	(2.8)	861	(2.8)	865	(2.8)	-0.00
40-0	38	(0.1)	28	(0.1)	10	(0.2)	-0.02	70	(0.1)	37	(0.1)	22	(0.1)	0.00
Missing	500	(1.6)	427	(1.7)	73	(1.3)	0.03	1,010	(1.6)	500	(1.6)	509	(1.6)	-0.00
Cancer stage, n (%)														
Stage 0	1,702	(5.5)	1,361	(5.3)	341	(6.1)	-0.03	3,413	(5.5)	1,702	(5.5)	1,711	(5.5)	-0.00
Stage Ⅰ	28,477	(91.2)	23,368	(91.2)	5,109	(91.3)	-0.00	56,952	(91.2)	28,477	(91.2)	28,475	(91.2)	0.00
Stage ⅡA	1,035	(3.3)	891	(3.5)	144	(2.6)	0.05	2,081	(3.3)	1,035	(3.3)	1,046	(3.4)	-0.00
Type of operation, n (%)														
VATS[b] Wedge Resection	5,820	(18.7)	4,519	(17.6)	1,301	(23.3)	-0.14	11,663	(18.7)	5,823	(18.7)	5,840	(18.7)	-0.00
VATS[b] Segmentectomy	4,692	(15.0)	3,711	(14.5)	981	(17.5)	-0.08	9,364	(15.0)	4,689	(15.0)	4,676	(15.0)	0.00
VATS[b] Lobectomy	19,579	(62.7)	16,595	(64.8)	2,984	(53.3)	0.23	39,188	(62.8)	18,582	(62.7)	19,606	(62.8)	-0.00
RATS[c] Lobectomy	1,123	(3.6)	795	(3.1)	328	(5.9)	-0.13	2,231	(3.6)	1,120	(3.6)	1,110	(3.6)	0.00
Complication at admission, n (%)														
Hypertension	7,770	(24.9)	6,400	(25.0)	1,370	(24.5)	0.01	15,482	(24.8)	7,760	(24.9)	7,723	(24.7)	0.00
Diabates	5,841	(18.7)	4,677	(18.3)	1,164	(20.8)	-0.07	11,666	(18.7)	5,840	(18.7)	5,826	(18.7)	0.00
Heart Failure	1,161	(3.7)	894	(3.5)	267	(4.8)	-0.06	2,294	(3.7)	1,158	(3.7)	1,136	(3.6)	0.00
Coronary Artery Disease	1,932	(6.2)	1,412	(5.5)	520	(9.3)	-0.15	3,851	(6.2)	1,932	(6.2)	1,919	(6.2)	0.00
Atrial Fibrillation/Atrial Flutter	1,058	(3.4)	735	(2.9)	323	(5.8)	-0.14	2,104	(3.4)	1,059	(3.4)	1,045	(3.4)	0.00
Interstitial Pneumonia	1,117	(3.6)	906	(3.5)	211	(3.8)	-0.01	2,239	(3.6)	1,118	(3.6)	1,121	(3.6)	-0.00
Chronic obstructive pulmonary Disease	2,237	(7.2)	1,880	(7.3)	357	(6.4)	0.04	4,440	(7.1)	2,236	(7.2)	2,203	(7.1)	0.00
Cirrhosis	181	(0.6)	142	(0.6)	39	(0.7)	-0.02	360	(0.6)	181	(0.6)	179	(0.6)	0.00
Demyelinating Disease	3	(0.0)	0	0	3	(0.1)	-0.03							
Cerebrovascular Disease	1,115	(3.6)	813	(3.2)	302	(5.4)	-0.11	2,261	(3.6)	1,121	(3.6)	1,140	(3.7)	-0.00
Renal Failure	587	(1.9)	434	(1.7)	153	(2.7)	-0.07	1,183	(1.9)	591	(1.9)	592	(1.9)	-0.00
Academic hospital, n (%)	8,249	(26.4)	6,801	(26.6)	1,448	(25.9)	0.02	16,616	(26.6)	8,257	(26.5)	8,358	(26.8)	-0.01
Oral medication at admission, n (%)														
Antiplatelets or anticoagulants	3,342	(10.7)	2,680	(10.5)	662	(11.8)	-0.04	6,682	(10.7)	3,342	(10.7)	3,340	(10.7)	0.00
One or more analgesics	2,720	(8.7)	2,222	(8.7)	498	(8.9)	-0.01	5,429	(8.7)	2,719	(8.7)	2,710	(8.7)	0.00
Intraoperative Narcotic Usage														
Fentanyl, mean (SD[d]), mg	0.65	(0.6)	0.65	(0.6)	0.66	(0.6)	-0.01	–	–	–	–	–	–	–
Remifentanyl, mean (SD[d]), mg	3.27	(2.0)	3.26	(2.0)	3.34	(2.0)	-0.04	–	–	–	–	–	–	–
Morphine, mean (SD[d]), mg	0.84	(8.0)	0.89	(8.3)	6.36	(1.4)	0.04	–	–	–	–	–	–	–
Ketamine, mean (SD[d]), mg	1.70	(16.8)	1.77	(17.7)	1.38	(12.0)	0.02	–	–	–	–	–	–	–
Postoperative Hospital Stay, mean (SD[d]), day	8.42	(9.6)	8.61	(10.0)	7.57	(7.5)	0.11	–	–	–	–	–	–	–
General Anaesthesia Time, n (%)														
0-120 min	1,674	(5.4)	1,385	(5.4)	289	(5.2)	0.01	–	–	–	–	–	–	–
121-240 min	14,119	(45.2)	11,544	(45.1)	2,575	(46.0)	-0.02	–	–	–	–	–	–	–
241-360 min	12.548	(40.2)	10.265	(40.1)	2,283	(40.8)	-0.02	–	–	–	–	–	–	–
360 < min	2,858	(9.2)	2,413	(9.4)	445	(8.0)	0.05	–	–	–	–	–	–	–
Missing	15	(0.1)	13	(0.1)	2	(0.0)	0.01	–	–	–	–	–	–	–
One or More Complication After Surgery, n (%)	2,038	(6.5)	1,577	(6.2)	461	(8.2)	-0.08	–	–	–	–	–	–	–

[a] Inverse Probability of Treatment Weighting

[b] Video-Assisted Thoracoscopic Surgery

[c] Robot-Assisted Thoracic Surgery

[d] Standard Deviation
