# Supplementary material for: Exploring the impact of perioperative analgesia on postoperative chronic analgesic prescriptions in patients with lung cancer undergoing minimally invasive thoracic surgery: A retrospective observational study
Source: Eur J Pain. 2024 Dec 27;29(2):e4774. doi: 10.1002/ejp.4774 (PMC11680971; doi:10.1002/ejp.4774)
Supplement: Supplementary file 4 [file EJP-29-0-s004.docx]

Table S3. The results of instrumental variable analysis

	OR	95% CI	*p* - value
Nerve Block	1	Reference	
Epidural Anaesthesia	1.07	0.94–1.22	0.306
Age			
< 60	1	Reference	
60–69	1.03	0.92–1.16	0.611
70–79	1.08	0.98–1.20	0.134
≧ 80	1.01	0.89–1.14	0.913
Sex (Female)	1.03	0.95–1.11	0.444
Body Mass Index, kg/m^2			
<18.5	1	Reference	
18.5–24.9	0.97	0.87–1.09	0.645
25.0–29.9	0.93	0.82–1.06	0.294
30.0–34.9	0.91	0.72–1.13	0.396
≧35.0	0.75	0.40–1.31	0.345
Missing	1.51	0.78–2.71	0.190
Charlson Comorbidity Index			
2	1	Reference	
3	0.98	0.90–1.07	0.617
≧4	1.00	0.87–1.15	0.953
Smoking	0.97	0.90–1.05	0.408
Barthel Index at Admission			
100	1	Reference	
95–45	0.97	0.79–1.18	0.797
40–0	0.91	0.27–2.31	0.865
Missing	1.06	0.82–1.35	0.638
Cancer Stage			
Stage 0	1	Reference	
Stage I	1.05	0.90–1.21	0.560
Stage IIA	1.01	0.80–1.27	0.917
Type of Operation			
VATS[a] Wedge Resection	1	Reference	
VATS[a] Segmentectomy	1.11	0.99–1.25	0.063
VATS[a] Lobectomy	1.13	1.03–1.25	0.008
RATS[b] Lobectomy	1.03	0.86–1.23	0.736
Complication at Admission			
Hypertension	1.00	0.93–1.07	0.949
Diabates	0.98	0.88–1.09	0.693
Heart Failure	0.85	0.70–1.03	0.099
Coronary Artery Disease	1.07	0.94–1.23	0.288
Atrial Fibrillation/Atrial Flutter	1.13	0.95–1.34	0.155
Interstitial Pneumonia	0.95	0.79–1.12	0.560
Chronic Obstructive Pulmonary Disease	0.98	0.85–1.12	0.749
Cirrhosis	1.06	0.68–1.58	0.780
Cerebrovascular Disease	1.06	0.89–1.27	0.462
Renal Failure	0.76	0.57–0.99	0.051
Academic Hospital	1.00	0.93–1.07	0.982
Oral Medication at Admission			
Antiplatelets or Anticoagulants	0.91	0.83–1.01	0.072
One or More Analgesics	2.97	2.73–3.23	<0.001
General Anaesthesia Time			
0–120 min	1	Reference	
121–240 min	0.96	0.83–1.12	0.576
240–360 min	1.08	0.93–1.26	0.300
> 360 min	1.39	1.17–1.65	<0.001
Missing	1.06	0.16–3.74	0.936

[a] Video-Assisted Thoracoscopic Surgery
[b] Robot-Assisted Thoracic Surgery
