# Supplementary material for: Exploring the impact of perioperative analgesia on postoperative chronic analgesic prescriptions in patients with lung cancer undergoing minimally invasive thoracic surgery: A retrospective observational study
Source: Eur J Pain. 2024 Dec 27;29(2):e4774. doi: 10.1002/ejp.4774 (PMC11680971; doi:10.1002/ejp.4774)
Supplement: Supplementary file 5 [file EJP-29-0-s005.docx]

Table S5. Results of logistic regression analysis in the Outpatients 6–12 M cohort following inverse probability of treatment weighting

Perioperative Pain Management	Proportion of Analgesic Prescription (%)	OR	95% CI	*p* - value
**Epidural Anaesthesia**	11.1	1.00	0.99 - 1.01	0.886
**Nerve Block**	10.9	1	Reference	
