# Supplementary material for: Exploring the impact of perioperative analgesia on postoperative chronic analgesic prescriptions in patients with lung cancer undergoing minimally invasive thoracic surgery: A retrospective observational study
Source: Eur J Pain. 2024 Dec 27;29(2):e4774. doi: 10.1002/ejp.4774 (PMC11680971; doi:10.1002/ejp.4774)
Supplement: Supplementary file 6 [file EJP-29-0-s006.docx]

Table S7. Results of the logistic regression analysis in the Outpatients 3–6 M cohort following inverse probability of treatment weighting with redefined analgesics (strong opioids)

	Proportion of Analgesic Prescription (%)	OR	95% CI	*p* - value
**Epidural Anesthesia**	0.3	1.00	1.00–1.00	0.321
**Nerve Block**	0.4	1	Reference	
