# Supplementary material for: Exploring the impact of perioperative analgesia on postoperative chronic analgesic prescriptions in patients with lung cancer undergoing minimally invasive thoracic surgery: A retrospective observational study
Source: Eur J Pain. 2024 Dec 27;29(2):e4774. doi: 10.1002/ejp.4774 (PMC11680971; doi:10.1002/ejp.4774)
Supplement: Supplementary file 7 [file EJP-29-0-s007.docx]

Table S6. Results of the logistic regression analysis in the Outpatients 3–6 M cohort following inverse probability of treatment weighting with redefined analgesics (NSAIDs[a], acetaminophen, weak opioids excluding codeine, strong opioids, pregabalin, mirogabalin, and neurotrophins)

	Proportion of Analgesic Prescription (%)	OR	95% CI	*p* - valu
**Epidural Anaesthesia**	11.0	1.01	0.99–1.03	0.811
**Nerve Block**	10.9	1	Reference	

[a] non-steroidal anti-inflammatory drugs
